# Performance evaluation of the Biograph mCT Flow PET/CT system according to the NEMA NU2-2012 standard

**DOI:** 10.1186/s40658-015-0132-1

**Published:** 2015-10-26

**Authors:** Ivo Rausch, Jacobo Cal-González, David Dapra, Hans Jürgen Gallowitsch, Peter Lind, Thomas Beyer, Gregory Minear

**Affiliations:** Quantitative Imaging and Medical Physics group (QIMP), Center for Medical Physics and Biomedical Engineering, Medical University of Vienna, Waehringer Guertel 18-20, 1090 Vienna, Austria; Universitätsklinikum Klagenfurt, Feschnigstraße 11, 9020 Klagenfurt, Austria; Division of Nuclear Medicine, Universitätsklinikum St. Pölten, Propst Führer-Straße 4, 3100 St. Pölten, Austria

**Keywords:** NEMA evaluation, Image quality, Biograph mCT Flow

## Abstract

**Background:**

The purpose of the study is to evaluate the physical performance of a Biograph mCT Flow 64-4R PET/CT system (Siemens Healthcare, Germany) and to compare clinical image quality in step-and-shoot (SS) and continuous table motion (CTM) acquisitions.

**Methods:**

The spatial resolution, sensitivity, count rate curves, and Image Quality (IQ) parameters following the National Electrical Manufactures Association (NEMA) NU2-2012 standard were evaluated. For resolution measurements, an ^18^F point source inside a glass capillary tube was used. Sensitivity measurements were based on a 70-cm-long polyethylene tube, filled with 4.5 MBq of FDG. Scatter fraction and count rates were measured using a 70-cm-long polyethylene cylinder with a diameter of 20 cm and a line source (1.04 GBq of FDG) inserted axially into the cylinder 4.5 cm off-centered. A NEMA IQ phantom containing six spheres (10- to 37-mm diameter) was used for the evaluation of the image quality. First, a single-bed scan was acquired (NEMA standard), followed by a two-bed scan (4 min each) of the IQ phantom with the image plane containing the spheres centered in the overlap region of the two bed positions. In addition, a scan of the same region in CTM mode was performed with a table speed of 0.6 mm/s. Furthermore, two patient scans were performed in CTM and SS mode. Image contrasts and patient images were compared between SS and CTM acquisitions.

**Results:**

Full Width Half Maximum (FWHM) of the spatial resolution ranged from 4.3 to 7.8 mm (radial distance 1 to 20 cm). The measured sensitivity was 9.6 kcps/MBq, both at the center of the FOV and 10 cm off-center. The measured noise equivalent count rate (NECR) peak was 185 kcps at 29.0 kBq/ml. The scatter fraction was 33.5 %. Image contrast recovery values (sphere-to-background of 8:1) were between 42 % (10-mm sphere) to 79 % (37-mm sphere). The background variability was between 2.1 and 5.3 % (SS) and between 2.4 and 6.9 % (CTM). No significant difference in image quality was observed between SS and CTM mode.

**Conclusions:**

The spatial resolution, sensitivity, scatter fraction, and count rates were in concordance with the published values for the predecessor system, the Biograph mCT. Contrast recovery values as well as image quality obtained in SS and CTM acquisition modes were similar.

## Background

Since the first PET/CT prototype became operational in 1998 [1], PET technology has advanced significantly, through, for example, the use of fully 3D acquisition mode and reconstruction [2], the adoption of fast lutetium oxyorthosilicate (LSO) crystals [3], which permit the use of shorter coincidence timing windows, the introduction of time of flight (TOF) [4–6], and the expansion of the axial field-of-view (FOV) for increased volume sensitivity [7]. The TOF measurement of two coincidence photons allows the localization of the positron annihilation point with a spatial accuracy that depends on the system timing resolution. This information can be used to significantly improve the image signal-to-noise ratio (SNR) of the reconstructed PET images, thus, allowing for better image quality and shorter acquisition times [8, 9]. The PET component of the Biograph mCT PET/CT system (Siemens Medical Solutions USA, Inc.) is able to incorporate TOF information into the reconstruction process [6].

**Fig. 1:**
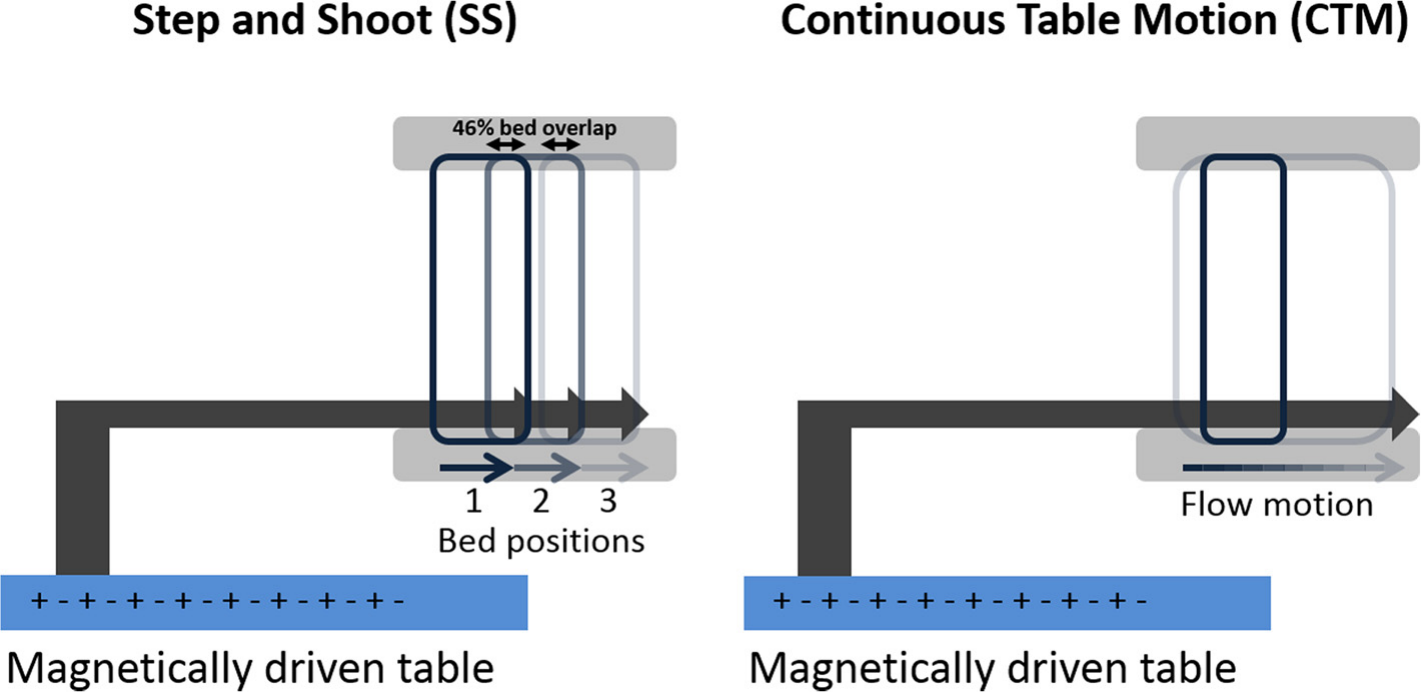
Schematic diagram of a step-and-shoot (*SS*) (*left*) and continuous (*right*) table motion (*CTM*) acquisition protocol. Within the SS protocol, the table is in a fixed position during an acquisition of data in the FOV and subsequently moves to the next position to acquire data of an axial range greater than the axial FOV of the system. In CTM, the table is moved continuously through the axial FOV of the scanner to acquire date of an extended scan range

Traditionally, whole-body PET examinations are performed in step-and-shoot (SS) mode, introducing the patient table motion between consecutive acquisitions of adjacent bed positions [10, 11]. For this acquisition protocol, planning and scanning are restricted by the fixed size of the detector array. An alternative to the SS acquisition mode is the continuous table motion (CTM) acquisition mode, recently implemented in the Siemens Biograph mCT Flow system (Siemens Medical Solutions USA, Inc.) [12]. Using this acquisition mode, the patient is moved through the gantry while PET emission data is continuously acquired. This technique was proposed by Dahlbom and colleges in 1992 [13] and integrated in a first PET-only system in 2001 [14]. A further implementation in a LSO-based PET/CT system was tested a few years later by several groups [15–17]. The advantage of the continuous bed motion is the increase of uniformity in the sensitivity profile across the axial FOV, due to the fact that the density of lines of response (LOR) does not depend of their axial position (as in the case of SS acquisitions) [13]. Figure 1 further illustrates SS and CTM acquisitions modes.

The evaluation of positron emission tomographs requires reproducible and reliable methods to allow the comparison of different systems using accepted measurement standards. The National Electrical Manufactures Association (NEMA) has published a series of procedures to evaluate the physical performance of PET systems [18, 19]. This NEMA standard is revised periodically, and the latest update of this publication resulted in the NEMA NU2-2012 standard [19].

The purpose of this work was to evaluate the physical performance of the Biograph mCT Flow 64-4R PET/CT system according to the NEMA protocol NU 2-2012 [19]. Furthermore, the clinical PET image quality was compared for standard SS and CTM acquisitions. Finally, the results of these measurements were compared with published values from its predecessor, the Biograph mCT system [6].

## Methods

### Siemens Biograph mCT Flow system

In this work, a new Biograph mCT Flow 64-4R PET/CT system (Siemens Medical Solutions USA, Inc.) was evaluated. The main feature of the system is the continuous motion of the patient table [12, 14], eliminating the need for overlapping bed positions between sequential acquisitions and independently defining arbitrary starting and end locations within the PET/CT imaging range. The system combines a 64-slice CT system (SOMATOM Definition AS+, Siemens Healthcare, Germany) with a whole-body LSO PET system. The PET component of the PET/CT is similar to its predecessors, the Biograph mCT [6] and the Biograph TPTV [7], with four detector rings of 842 mm in diameter, each consisting of 48 detector blocks and each block containing an array of 13 × 13 crystals (4 mm × 4 mm × 20 mm). This configuration provides an axial field-of-view (FOV) of 22.1 cm, which results in 109 image planes (slice thickness of 2.027 mm) when using SS acquisition mode. The patient bore is 78 cm and identical to the previous mCT system. Sinograms comprise 400 radial and 168 angular bins, 621 total slices (109 direct and 512 oblique planes), and 13 TOF bins (312 ps wide) [6]. The default energy window is 435–650 keV and the default coincidence and TOF timing resolution windows are 4.066 and 0.555 ns, respectively.

The CTM (FlowMotion) technology requires a new design of the patient table and the acquisition electronics. The patient handling system (PHS) contains a horizontal magnetic drive system that enables a continuous table motion with a positioning accuracy of less than 0.25 mm. Table speeds range from 0.1 to 200 mm/s, sufficient to address the needs of both PET and CT [12]. Similar to CT acquisitions, the FlowMotion acquisition system tracks the bed position in real time and stores this information in the listmode data file as a new tag for each coincidence event [20]. The extra bed position information is then employed during the data processing, thus, assuring that the final reconstructed images contain the correct position information. The acquisition electronics is based on solid-state components capable of continuously recording and storing detector addresses. A new electronic buffer was designed to support higher counting rates during data acquisition [21], which is connected to solid state drives (SSDs) with capability to store up to 30 × 10^9^ coincidence events [22].

### NEMA NU 2-2012 measurements

All measurements were performed at the site of the clinical installation of the system between August 2014 and December 2014.

#### Spatial resolution

As recommended by the NEMA NU 2-2012 protocol [19], an ^18^F-FDG point source (size <1 × 1 × 1 mm) was used inside a 75-mm-long glass capillary tube (Hirschmann Laborgeräte, Hämatokrit-Kapillaren, REF 9100160) with an inner diameter of 0.9–1.0 mm and a wall thickness of 0.4 mm. The total activity was low enough to keep dead time losses and randoms below 5 % of the total events, as suggested by NEMA NU2-2012. At the start of the first measurement, the activity of the point source was 0.62 MBq. Data were acquired at three transaxial locations(*x*, *y*): (1,0), (10,0), and (20,0) and at two axial positions (*z*) within the PET FOV: center FOV and three-eighth off-center FOV. At least 2 × 10^6^coincident events were acquired at each position. TOF information was not used in this measurement. The acquired sinogram data were Fourier rebinned (FORE) and reconstructed with filtered backprojection (FBP) into a 400 × 400 × 109 matrix with 2 × 2 × 2-mm pixel size. No attenuation and scatter correction and no post-smoothing filter were applied. The Full Width Half Maximum (FWHM) and the Full Width Tenth Maximum (FWTM) of the reconstructed point-spread functions (PSF) were obtained from these images following the NEMA NU2-2012 protocol using in-house software.

#### Sensitivity

A 70-cm-long polyethylene tube (inner diameter 1 mm; outer diameter 3 mm) was used, filled with 4.5 MBq of FDG (at start of acquisition) and placed inside of five concentric aluminum sleeves of the same length with known diameters [19]. For each aluminum sleeve, data was acquired for 300 s (11.9 × 10^6^ prompts in first acquisition). The measurements were performed at the center of the FOV and at 10-cm radial offset. Online random subtraction was applied from a delayed coincidence window. The corrected true coincidence count rate was recorded as a function of sleeve thickness and extrapolated to a zero thickness sleeve. The system sensitivity was then computed as the ratio between the true count rate with no absorption and the starting activity. The data were analyzed using the NEMA NU2-2012 protocol using in-house software.

#### Scatter fraction and count rate performance

The phantom used for both measurements was a 70-cm-long polyethylene cylinder with a diameter of 20 cm, with a line source inserted axially into the cylinder 4.5 cm radially from the center. The line source was filled with an initial activity of 1.04 GBq of ^18^F, in order to achieve count rates beyond the expected peak of the noise equivalent count rate (NECR) [6, 7]. Data were acquired for more than 15 h resulting in 45 frames, each with 600-s acquisition time and an inter-frame delay of 600 s. Online random subtraction was applied from the delayed coincidence window to account also for the intrinsic LSO radioactivity [23]. The scatter fraction and NECR were determined as described in the NEMA NU2-2012 standard [19] acquired using software tools provided by the manufacturer and evaluated using in-house software. System true event rate, random event rate, scatter event rate, NECR, and the scatter fraction are reported.

#### Accuracy of count losses and random corrections

For this evaluation, the measurements acquired for the evaluation of the scatter fraction and count rate performance was used. The data was corrected for dead time, scatter, and random according to the NEMA NU2-2012 protocol and reconstructed using the FORE FBP algorithm. Of note, the NEMA NU2-2012 protocol specifies to use the standard whole-body algorithm [19]. The choice of the FBP algorithm was done to stay comparable with the vendors’ specifications and published values [24]. Evaluation was done using in-house software. The axial end slices (first and last slice) were excluded from the analysis. Maximum modulus count rate error at peak NECR as well as maximum and minimum error for all activity concentrations are reported.

#### Image quality

A NEMA image quality [19] phantom containing six spheres with internal diameters of 10, 13, 17, 22, 28 and 37 mm was used for the evaluation of the image quality. A cylindrical insert with a diameter of 5 cm containing a low density material with an average density of 0.3 g/ml was positioned in the center of the phantom to simulate lung tissue and provide a non-uniform attenuation distribution. This phantom was filled with a solution of water and ^18^F-FDG containing a background activity concentration of ~5.3 kBq/ml. The four smallest spheres were filled with a target-to-background ratio (TBR) of 8:1 (first set of scans) and 4:1 (second set of scans). The remaining two largest spheres were filled with non-radioactive water. The phantom was positioned with all spheres aligned within the same transaxial image plane in the center of the FOV. To simulate a clinical situation with activity outside the FOV, the cylindrical phantom used for the count rate measurement was placed besides the Image Quality (IQ) phantom as described in NEMA NU2-2012. The line source for the scatter phantom was filled with a solution of ^18^F-FDG and water. The activity in the line source of the scatter phantom was 100 and 99 MBq at the start of the first and second set of scans, respectively. Three sequential measurements of 240 s each were acquired for a single-bed position subsequent to a CT transmission scan (tube voltage 120 kVp, tube current 289 mA, matrix 512 with 1.5-mm pixel size, pitch 0.8) for attenuation correction. All data were corrected for random coincidences, normalization, dead time losses, scatter, and attenuation. Data were reconstructed with an ordered-subset expectation maximization (OSEM) [25] 3D iterative algorithm, using 2 iterations (i) and 24 subsets (s), and additionally with 2 iterations and 21 subsets applying PSF correction and TOF. For both reconstructions a 200 × 200 matrix size was used and a post-reconstruction Gaussian filtering with 3-mm FWHM was applied.

NEMA NU2-2012 standards were followed to evaluate the image quality. The average (and range) percent contrast obtained for hot and cold spheres, the average (and range) standard deviation of the background counts, and the mean residual error in scatter and attenuation corrections were evaluated.

### Comparison of sequential and FlowMotion acquisition modes

Subsequent to the image quality measurement, a two-bed scan (4 min each) of the IQ phantom were acquired with the image plane containing the spheres centered in the overlap region of the two bed positions (bed overlap 46 %). A second scan of the same region in CTM mode was acquired at a table speed of 0.6 mm/s. The table speed was selected to cover an axial FOV of a standard five-bed scan (a common clinical axial FOV) in the same time as the SS mode. The order (CTM after SS) was reversed for the 4:1 TBR to account for potential issues related to differences in background activity in the two scans. Reconstructions were performed with an OSEM [25] 3D iterative algorithm with 2i and 21 s applying PSF as well as TOF and using a 200 × 200 matrix. Images were post-filtered with a 3-mm FWHM Gaussian filter. The image contrast and the background variability for the six spheres in SS and CTM acquisitions was compared by means of a paired students’ *t* test using the software R (R Foundation for Statistical Computing, Vienna, Austria). Furthermore, the accuracy of scatter and attenuation corrections in both acquisition modes was evaluated. The contrast values, background variability, and the accuracy of attenuation and scatter correction for the lung insert were evaluated with an in-house developed plug-in for ImageJ (U. S. National Institutes of Health, Bethesda, Maryland, USA), which had been previously validated and used during two Austrian PET Inter-Laboratory comparison studies [26, 27].

#### Whole-body patient scans

For comparison of the impact on image quality in clinical routine, two patients who underwent routine FDG examinations (~300 MBq FDG, ~60 min pi) were scanned subsequently, in CTM and SS mode. The CTM scans were acquired with a table speed of 1.0 mm/s, while the SS acquisitions were performed with an acquisition time of 2.3 min per bed with a bed overlap of 46 %. These settings were selected to cover the same axial FOV in the same time as performed with the IQ phantom measurements. One CT acquisition was acquired for attenuation correction with on-site clinical parameters (tube potential 120 kVp, modulated tube current 120 mAs reference, pitch 1.1) and used to correct both (SS and CTM) PET acquisitions. Images were reconstructed with an OSEM 3D algorithm (2i, 21 s) with PSF modeling and TOF information and a 3-mm Gaussian post-filtering. Whole-body images were compared visually by two experienced imaging specialists (one nuclear medicine physician and one imaging physicist). Mean standardized uptake values (SUVs_mean_) and coefficient of variations (CVs) were calculated for spherical volumes of interest (VOI) with 3-cm diameter placed in the liver and the bladder. In addition, a region of interest (ROI) of about 110 cm^2^ was positioned upon the last visible parts of the legs in order to evaluate noise at the extreme end of the axial scan range.

## Results

### Spatial resolution

The transverse and axial resolutions for the different positions of the point source are summarized in Table 1 that lists FWHM and FWTM values at 1, 10, and 20 cm. For comparison, the NEMA NU2-2007 [18] values for its predecessor (Biograph mCT) are included. No noticeable differences were found between the two systems.

**Table 1:** Spatial resolution measured for the PET component of the mCT Flow system (NEMA NU2-2012) and comparison with the published values (NEMA NU2-2007) of its predecessor, the mCT system [6]

Spatial resolution	Distance (cm)	Measured (mCT Flow)		Published (mCT) [6]	
		FWHM (mm)	FWTM (mm)	FWHM (mm)	FWTM (mm)
Transverse	1	4.33	8.60	4.4 ± 0.1	8.6 ± 0.1
Axial	1	4.25	8.55	4.4 ± 0.1	8.7 ± 0.2
Transverse radial	10	5.16	9.30	5.2 ± 0.1	9.4 ± 0.1
Transverse tangential	10	4.72	9.68	4.7 ± 0.1	9.2 ± 0.1
Axial	10	5.85	11.06	5.9 ± 0.1	10.9 ± 0.3
Transverse radial	20	5.55	9.84	–	–
Transverse tangential	20	6.48	12.68	–	–
Axial	20	7.80	13.7	–	–

### Sensitivity

**Fig. 2:**
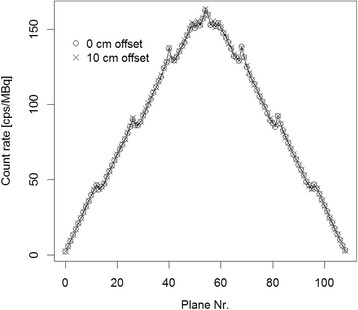
mCT Flow. Axial sensitivity profile for the measurements with the line source in the center of the field of view and at 10-cm radial offset

The mCT Flow system had a sensitivity of 9.6 kcps/MBq for both, the 0- and 10-cm off-center position. The axial sensitivity profiles with the line source placed at the center of the FOV and 10-cm radial offset are shown in Fig. 2.

### Scatter fraction and count rate performance

**Fig. 3:**
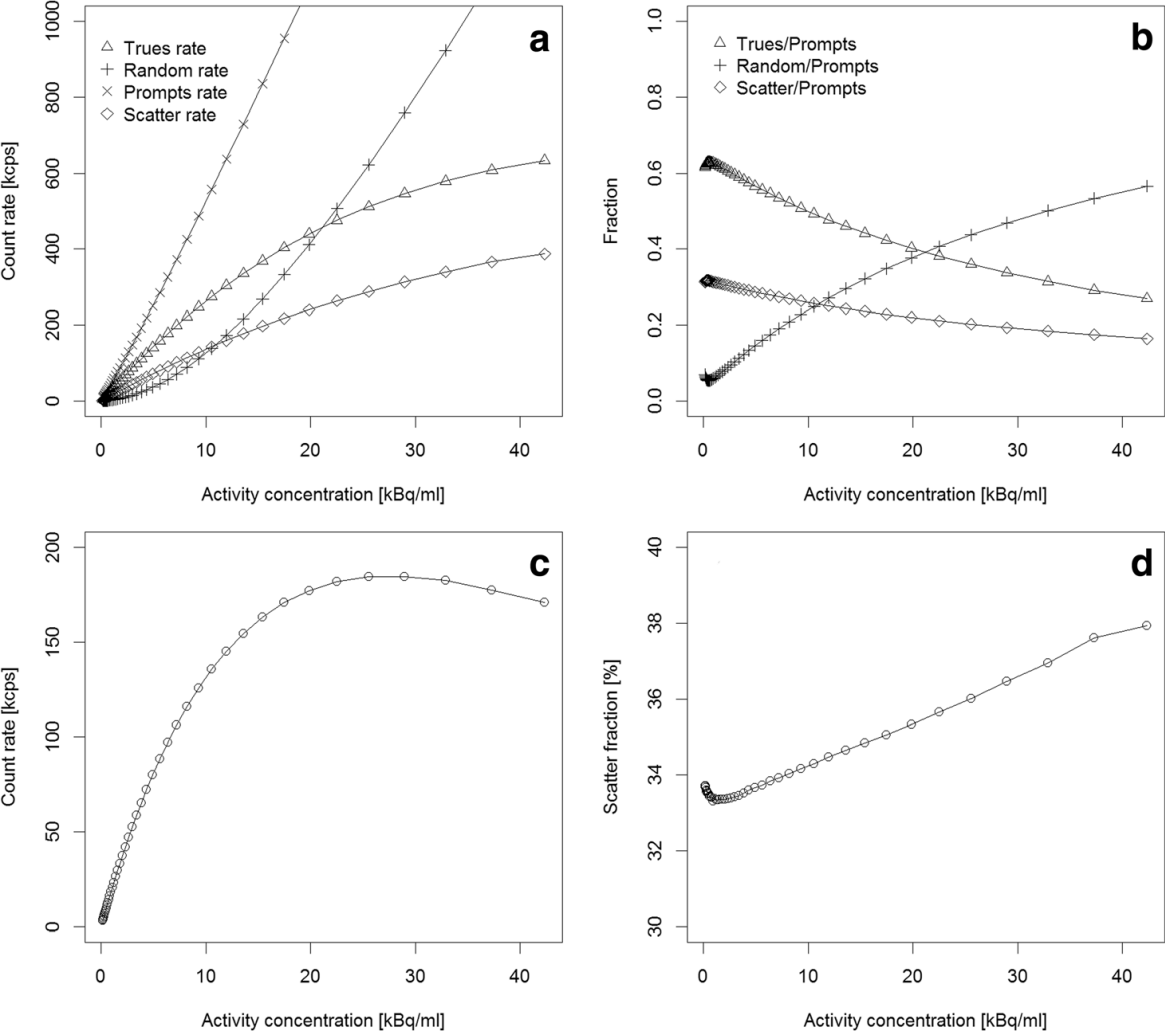
mCT Flow. **a** Prompts, trues, randoms, and scatter count rates. **b** Fraction of trues, randoms, and scatter counts vs the total number of coincidences processed. **c** NECR curve for the measured range of activities. **d** Scatter fraction (in %) for the same range of activities

The peak NECR (*k* = 0) was 185 kcps at 29.0 kBq/ml. The scatter fraction was calculated to be 33.5 % at the minimum randoms to prompt ratio [19], which corresponded to 0.51 kBq/ml and 33.4 % at the peak NECR activity concentration. The NECR peak and the scatter fraction obtained for the Biograph mCT Flow system are compared to its predecessor, the Biograph mCT in Table 2. No noticeable difference between the two systems was observed. Plots of the trues, randoms, and scatter event rates as well as the NECR and the scatter fraction curves as a function of activity are shown in Fig. 3.

**Table 2:** Measured count rates and scatter fraction for the mCT Flow system (NEMA NU2-2012) and comparison with the published values (NEMA NU2-2007) of the mCT system [6]

Parameter	Measured (mCT Flow)	Published (mCT) [6]
Peak true rate	634 kcps at 42.4 kBq/ml	–
Peak NECR	185 kcps at 29.0 kBq/ml	(180 ± 8) kcps at (28.3 ± 0.6) kBq/ml
Scatter fraction at low counting rates	33.5 % at 0.5 kBq/ml	33.2 ± 0.7 %
Scatter fraction at Peak NECR	33.4 % at 29 kBq/ml

### Count rate accuracy

**Fig. 4:**
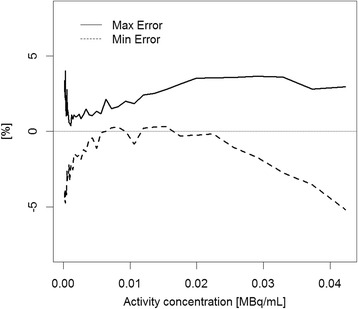
Maximum (*solid line*) and minimum (*dashed line*) relative count rate error for the different activity distributions. The first and the last slice of the acquisitions were excluded from this evaluation

The relative count rate error at the activity concentration of the NECR peak (29.0 kBq/ml) was 3.7 %. The maximum and minimum errors for all activity concentrations are depicted in Fig. 4.

### Image quality phantom (hot spheres at the center of the axial FOV)

The calculated activities at the start of the second scan were 5.2 and 5.3 kBq/ml for the 8:1 and 4:1 TBR, respectively. The contrast, background variability, and average residuals in the lung computed from these images are shown in Table 3. As in the previous section, all the measurements were performed with a single PET FOV (corresponds to the SS acquisition protocol) [19].

**Table 3:** Image quality results for 4:1 and 8:1 sphere-to-background ratio according to the NEMA NU2-2012 standard

Sphere diameter (mm)	Sphere-to-background ratio = 4:1				Sphere-to-background ratio = 8:1			
	Contrast recovery (%)		Background variability (%)		Contrast recovery (%)		Background variability (%)	
	OSEM mean (max/min)	PSF + TOF mean (max/min)	OSEM mean (max/min)	PSF + TOF mean (max/min)	OSEM mean (max/min)	PSF + TOF mean (max/min)	OSEM mean (max/min)	PSF + TOF mean (max/min)
10	21.5 (19.1/24.9)	28.5 (24.3/35.4)	5.6 (5.2/5.9)	4.7 (4.0/5.4)	35.3 (33.9/37.7)	42.4 (41.1/45.1)	5.7 (4.9/6.4)	5.5 (5.3/5.6)
13	33.4 (32.7/34.6)	42.3 (41.3/43.9)	4.8 (4.3/5.2)	4.3 (3.7/4.8)	51.5 (49.3/54.1)	63.6 (61.3/65.2)	5.0 (5.6/5.4)	4.8 (4.6/5.0)
17	47.8 (46.2/49.7)	58.4 (56.6/60.1)	3.9 (3.6/4.3)	3.7 (3.4/4.0)	59.6 (58.3/61.3)	68.9 (68.1/69.3)	4.1 (4.0/4.4)	4.1 (3.7/4.3)
22	62.1 (59.7/64.3)	71.7 (67.8/73.6)	3.2 (3.0/3.5)	3.4 (3.2/3.6)	69.9 (68.8/71.2)	76.7 (76.1/77.1)	3.4 (3.4/5.6)	3.4 (3.1/3.7)
28	61.2 (60.4/61.8)	70.1 (69.6/71.0)	2.7 (2.6/3.0)	3.2 (3.1/3.2)	61.8 (59.4/64.4)	70.6 (69.3/71.6)	2.8 (2.6/3.0)	2.9 (2.6/3.1)
37	67.9 (65.7/69.3)	78.3 (76.3/80.2)	2.3 (2.2/2.4)	3.0 (2.9/3.1)	68.2 (68.0/68.6)	78.5 (78.2/78.7)	2.1 (1.9/2.4)	2.5 (2.3/2.6)
Lung residual % (σ)			22.8 (1.6)	12.7 (1.4)			22.3 (1.5)	12.2 (1.2)

### Image quality phantom (hot spheres between two bed positions)

**Fig. 5:**
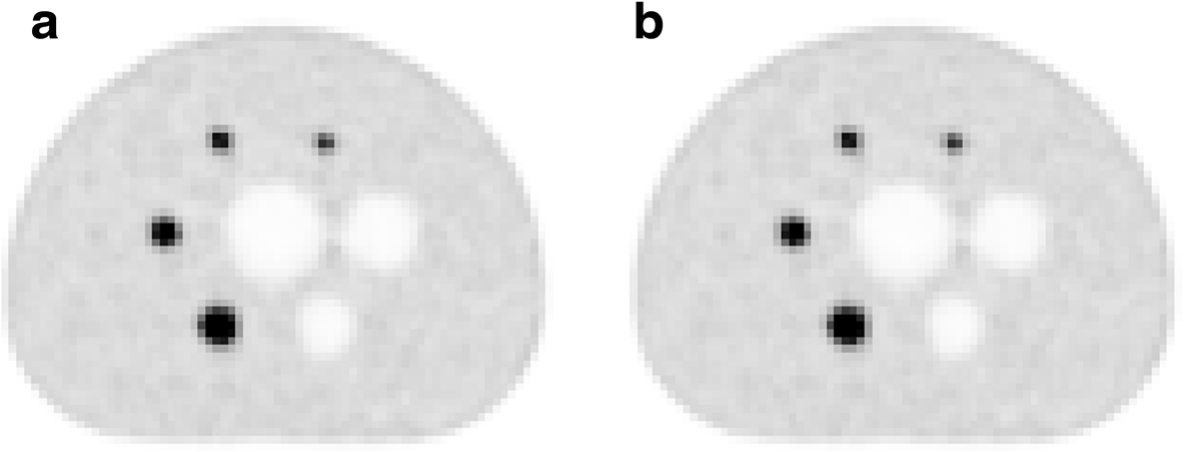
Central slice of the image quality phantom for sequential (**a**) and CTM (**b**) acquisition modes. The sphere-to-background ratio is 8:1. The CTM acquisition followed the sequential subsequently

The comparison of the contrast, background variability, and average residuals in the lung with SS and CTM acquisitions is presented in Table 4 for the 4:1 and the 8:1 TBR. The reconstructions of the transaxial sections through the center of the spheres within the image quality phantom for SS and CTM mode are shown in Fig. 5 for the scan of the 8:1 sphere-to-background activity ratio. No significant difference in CRCs was found between SS and CTM acquisitions (*p* = 0.35). The difference in background variability, however, was significant between both acquisition modes (*p* < 0.01).

**Table 4:** Image quality comparison for SS and CTM acquisition modes. The sphere-to-background ratio is 4:1 and 8:1. For the 4:1 ratio, the SS acquisition followed the CTM acquisition subsequently. For the 8:1 ratio, the CTM acquisition followed the SS subsequently

Sphere diameter (mm)	Sphere-to-background ration = 4:1				Sphere-to-background ration = 8:1			
	Contrast recovery (%)		Background variability (%)		Contrast recovery (%)		Background variability (%)	
	SS	CTM	SS	CTM	SS	CTM	SS	CTM
10	26.0	28.3	5.3	6.9	43.7	41.9	4.9	6.3
13	44.8	47.9	4.6	6.8	59.8	63.1	4.1	5.4
17	61.9	58.4	3.9	5.6	66.6	68.1	3.3	4.4
22	69.2	70.8	3.1	4.7	75.5	76.6	2.8	3.6
28	68.8	67.0	2.5	3.8	70.7	71.3	2.5	3.0
37	76.5	76.9	2.2	2.9	77.5	77.7	2.1	2.4
Lung residual (%) (σ)			12.2 (0.9)	12.3 (1.0)			12.3 (0.9)	12.1 (0.6)

### Patient studies

Two female patients (age 74 and 52 years, weight 61 and 57 kg) were imaged on the mCT Flow following the CTM and SS protocols. In both cases, the delay between the start of the CTM and SS measurements was 17 min, which resulted in an overall decreased activity of 10 % during the course of the two scans due to the physical decay of ^18^F.

**Fig. 6:**
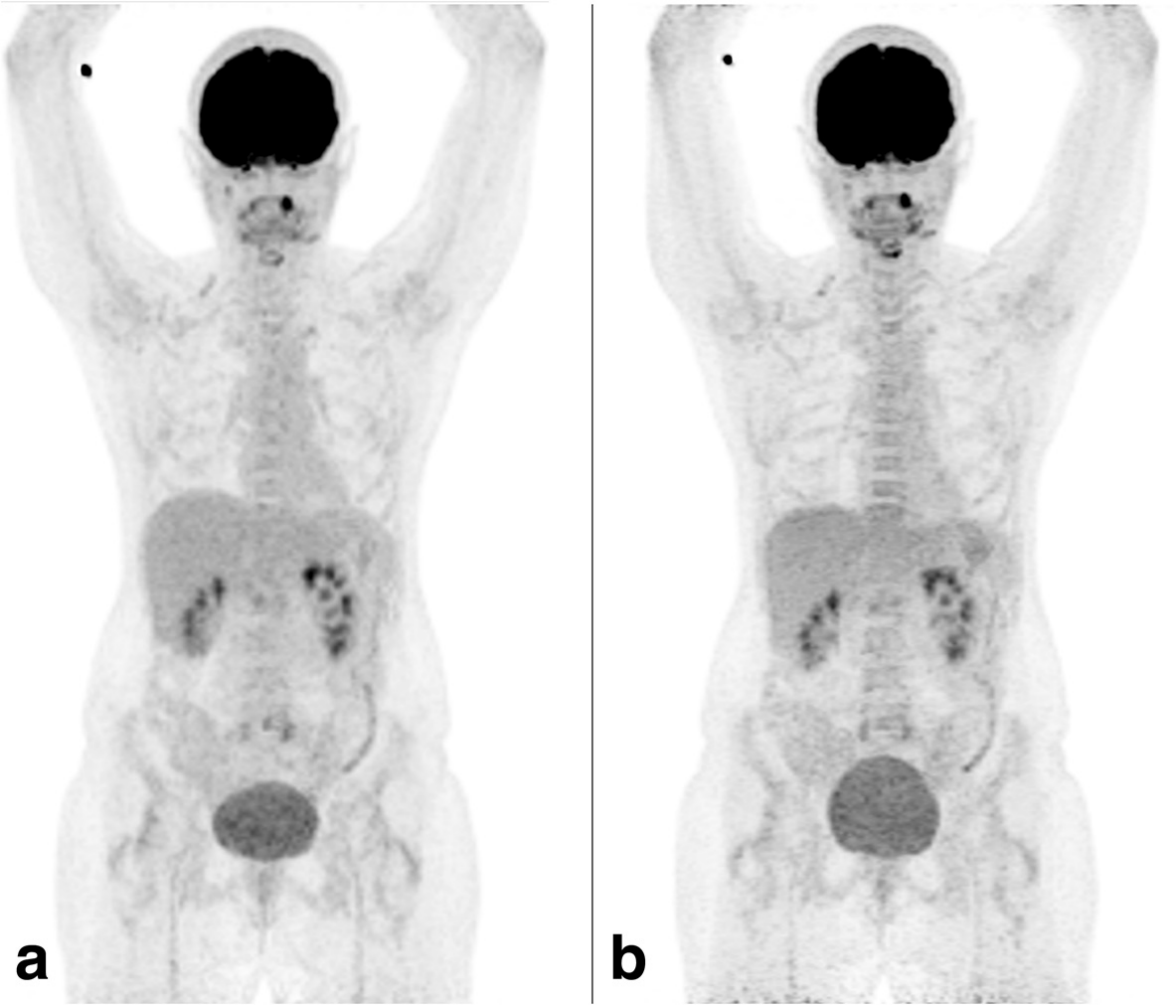
MIP of a patient scan acquired in **a** CTM and **b** SS mode. The overall activity concentration in **b** is about 10 % less due to radioactive decay between the two scans. Image quality is very similar. The changes in bladder filling are caused by the time between the scans. Small differences in local uptake (e.g., larynx) are supposed to be caused by continuous physiological tracer uptake

No visual difference in overall PET image quality was observed by two experienced imaging experts. Nonetheless, increased noise was observed in the images acquired in SS mode (Fig. 6) at the cranial and lower extremities limits of the axial scan range. The SUV and CV values for the reference regions are given in Table 5. For the ROIs placed in the end planes, SUV_mean_ values were similar for both acquisitions but CVs changed from 75.0 and 50.0 % (CTM) to 96.6 and 65.5 % (SS), respectively.

**Table 5:** SUV_mean_ values and CVs of reference regions in the two patient scans. The values are given for each patient separately for both, the SS and the CTM mode

Patient 1/patient 2	CTM–SUV (g/ml)	SS–SUV (g/ml)	CTM–CV (%)	SS–CV (%)
Liver	2.0/2.1	1.9/2.2	9.2/11.3	10.8/11.8
Bladder	9.2/3.7	5.5/3.5	10.8/11.0	8.6/9.3
Edge ROI	0.3/0.5	0.3/0.6	75.0/50.0	96.6/65.5

## Discussion

Our data indicate a similar performance of the PET components of the mCT Flow and its predecessor system, the mCT [6] with the residual differences being discussed below. Image quality, as assessed by the standard IQ phantom [19] and sample oncology patients, was also similar for the CTM and SS acquisitions with the exception of slightly increased noise levels for the SS image planes at the edge of the axial FOV (Fig. 6).

Of note, there are no published performance measurements for the mCT system series based on the most recent NEMA NU2-2012 standard. The data published for the mCT [6] are based on the older NEMA NU-2007 standard [18]. The most significant difference between both standards is the change of the positions of the point source for spatial resolution measurements. NEMA NU2-2012 requires a measurement at 1-, 10-, and 20-cm radial offset position from the isocenter of the PET FOV while 2007 version requires a single measurement at 1 cm and two measurements (in *x* and *y* direction) at 10-cm offset. Furthermore, the axial placement of the off-center axial point sources was changed from one fourth of the axial FOV from the center to three eighth of the axial FOV from the center, respectively. Of note, the 2012 standard permits for a range of image reconstruction methods. Thus, users may opt for applying PSF and other geometric corrections to the data, which inevitable makes inter-system comparisons more difficult.

For the measurements for the sensitivity, the count rate performance, and the associated relative count rate error, a provision was made in NEMA NU2-2012 for elevating the phantoms, if the vertical range of the patient table is insufficient for the required placement of the phantoms. Furthermore, the filling tolerance was expanded and a method for correcting the measured activity concentration for axially extended in-tube activity was introduced. Nonetheless, here, the patient handling system had a sufficient vertical table range and the line sources were filled to meet exactly the 700-mm in-tube filling, and, therefore, these changes were not applicable to our measurements.

The NEMA NU2-2012 standard requires a correction for the duration of the acquisition for the sensitivity test. With the used duration (300 s) and ^18^F as isotope, this corresponds to a change of 1.6 % in outcome, which is negligible.

The count rate error evaluation was also slightly modified in the 2012 standard. The expected value of the true count rate is gained by a fit through all data below the peak NECR (2012 standard) as opposed to the extrapolated values from the last three acquisitions (2007 standard). While the difference in error may be small, the exclusion of the end slices as permitted in the NEMA NU2-2012 protocol should be followed anyway, as these end slices often suffer from high noise levels due to the low sensitivity of the scanner at the end of the axial FOV (Fig. 2).

Despite the modifications from the 2007 to the 2012 standard, the results for the system sensitivity, the count rates, NECR, scatter fraction, corrections for count losses and random measurements as well as the count rate error are in accordance with published values for the mCT system [6] following the NEMA NU2-2007 standard.

Regarding the IQ test, the only difference between the 2007 and 2012 standard is a reduction of emission scan time by a factor of 2. The contrast recoveries for the “OSEM” reconstructed image were in accordance with results gained in similar systems [6, 7]. The use of PSF reconstruction with TOF information resulted in an overall improvement in contrast recovery (CRC) (Table 3). Nevertheless, contrast recoveries for both reconstructions were substantially lower than the values published for the mCT system [6].

This difference warrants further discussion. A possible explanation could be the differences in reconstruction algorithms and post-filtering used in [6] and here. Previous studies have shown that by incorporating PSF modeling into the image reconstruction, an overestimation of the CRC can be observed, in particular, when no post-filtering is applied [25, 28, 29]. This can be attributed in part to the generation of Gibbs artifacts at the edge of the reconstructed object, which are particularly noticeable in smaller objects [25, 29]. These artifacts can be reduced by applying post-filtering with small to modest amounts of blurring but at the cost of lower image contrast. When imaging a slightly modified NEMA IQ phantom on a Biograph mCT system, Armstrong and colleagues demonstrated an overcorrection of the SUV_max_ of up to 151 % for the 13-mm sphere in case no post-filtering was applied [25]. After applying a Gaussian filter (FWHM = 2.9 mm), this overestimation was reduced to 138 %.

In a study by Tong et al. using the same system, the authors demonstrated a reduction of 5–8 % of the mean CRC for the 10- to 22-mm spheres at a TBR of 4:1 after a 4-mm Gaussian post-filter was applied to data reconstructed with 3D OSEM + PSF but without TOF [30]. Based on the results of Armstrong [25], this difference would increase for the smaller spheres when TOF was included into the reconstruction algorithm. In comparison, Marti-Climent et al. [24] obtained CRC similar to the data here, with a standard IQ phantom and a TBR of 4:1 following 3D-OSEM reconstruction with PSF and TOF and a 2-mm Gaussian post-filter. These studies clearly demonstrate the dependence of CRC on post-filtering and provide one explanation to any observed differences in CRC across centers using the same PET/CT system.

Furthermore, the 50 % reduction in acquisition time, as suggested in the 2012 standards, resulted in a higher inter-scan variance of the mCT Flow quality results compared to the data quoted in [6]. The spread of the CRC from the three sequential measurements was rather high, especially for the 10-mm sphere with a standard deviation of ±5.6 %.

Lesion detectability depends on the acquisition time [31]. Using a whole-body phantom with simulated hot lesions of 8 to 16 mm in diameter, Kadrmas and colleagues demonstrated that lesion detectability is a function of both scan time and post-filtering. This relationship was modeled from an empirical logarithmic relationship. Using a model numerical observer method to calculate localized receiver operator curves (LROC), the authors measured a reduction of approximately 16 % in the area under the ROC (AROC) when the acquisition time was reduced from 240 to 120 s per PET FOV from OSEM + PSF + TOF reconstructed data with a subsequently applied Gaussian post-filter of about 1.3 voxels, or less. Based on this relationship, a considerable decrease in detectability is expected when the acquisition time is reduced by a factor of 2, which is inevitably related to image contrast. In our measurements, the acquisition duration was 240 s instead of 600 s as used by Jakoby et al. [6]. Furthermore, the reconstruction parameter differed from the ones in [6] (3D OSEM (3i, 24 s) and 3D OSEM (2i, 21 s + PSF + TOF), both with 3-mm FWHM Gaussian post-filtering in this study vs 2D OSEM (3i, 8 s) with 5-mm Gaussian post-filtering and 3D OSEM (2i, 21 s + PSF + TOF) with no post-filtering in [6]). When the effects of post-filtering and acquisition duration are taken into account, the values measured in this study show a closer conformance to those previously published [6].

Background variability values were slightly lower for the PSF + TOF reconstruction compared to the published values from the mCT system, which may be related to using a 3-mm Gaussian post-filtering. For the OSEM reconstruction, the results were slightly worse, which can be explained by the difference in applied post-filtering (3-mm FWHM vs 5-mm FWHM) and the difference in iterations and subsets; the larger number of subsets in our study will lead to an enhancement of the noise in the image. Nevertheless, the choice of the reconstruction parameters in our study for the evaluation of the image quality was based on the NEMA NU2 standard that asks to use clinically relevant settings. For the purpose of comparison between systems, this may not be sufficiently accurate. The clinically used settings of each system strongly depend on local on-site preferences. Therefore, it is suggested to define a set of parameters (iterations, subsets, post-filtering, and algorithm) for each system for a standardized evaluation of the image quality. This set of parameters should be defined by the vendor and be delivered with the system, e.g., within the manual.

Finally, no significant differences in the measured IQ was observed for sequential and for CTM acquisition modes (Table 4). The CRCs were similar in both acquisition modes. This is most probably a result of the bed overlap of 46 % used in SS mode. Dahlbom et al. [32] stated that a similar noise reduction as with CTM is achieved with overlapping bed positions. Nevertheless, Brasse et al. [15] reported improvements in contrast of 16 to 45 % with CTM when placing an IQ phantom in the bed overlap region. However, this study was performed on a different system with an axial FOV of 15.2 cm with a 4-cm bed overlap and, thus, rendering a comparison a challenge. The only difference between the CTM and SS mode was a slightly elevated background variability in CTM mode which was statistically significant for the IQ phantom measurements. A similar increase was observed in the CVs (which equate to the background variability) in the bladder of the two patients. The bladder was chosen to test for this issue as it contains a uniform activity concentration and therefore similar to the background compartment in the IQ phantom.

The elevated background variability is assumed to be caused by the choice of the table speed and the data processing techniques specific to CTM acquisitions. The table speed was estimated to cover a standard FOV in approximately the same time in CTM and SS mode. For example, for a five-bed position acquisition, this is total axial FOV = axial FOV + 4 × axial FOV × 0.54 = 69.8 cm were the 0.54 is accounting for the bed overlap. With a total scan time of 5 × 4 min, this corresponds to a table speed of 0.58 mm/s. For an axial FOV excluding the low sensitivity regions at the axial endpoints of the scan range (a hypothetical scan with an unlimited number of bed positions), a 4-min acquisition per bed position corresponds to a table speed of 0.5 mm/s (this table speed is also recommended in [12] for a similar image quality). In this study, the IQ phantom images were acquired with a table speed of 0.6 mm/s in CTM mode. Therefore, the CTM acquisitions resulted in a lower number of collected true events than in SS mode, which in turn resulted in an elevated background variability [33]. Nevertheless, based on the findings of Molina-Duran and colleges, [33] not the entire increase in BG variability can be explained from the decrease in count density. This is further supported by the findings of Braun and colleges [34] who showed increased noise in CTM acquisitions in comparison to SS acquisitions with equivalent numbers of true events. Therefore, we suspect at least a part of the increase is caused by noise added during the subtraction of delayed sinograms due to difficulties in creating a mean random sinograms in CTM mode, the normalization technique [35] and/or the multiple interpolation steps implemented in the “on the fly rebinning” of the projection data [22].

There were no visual differences in image quality of the patient scans (Fig. 6). Furthermore, no relevant difference of the SUV_mean_ of the liver as reference organ could be observed, and the comparison of the CVs of the liver showed no clinical relevant differences. Moreover, increases in CV using the CTM mode could not be reproduced within these VOIs and is most probably attributable to physiological changes. This and the IQ phantom tests indicate the equivalence of the SS and CTM acquisition mode for clinical purposes. Nevertheless, enhanced noise at the axial edges in the SS mode acquisition was observed. This was further confirmed by the quantitative evaluation of the CV from a corresponding region which showed an average increase in the CV of 30 % for the SS acquisitions. This is the result of the low sensitivity in this part of the axial FOV (Fig. 2) and is avoided using the CTM technology [32].

Nevertheless, CTM enables the use of varying table speeds for different axial regions. This can be used to optimize the protocols, e.g., a faster feeding over the legs to lower overall scan time or to adjust noise properties in regions of interest, e.g., slower feeding in the abdominal region. Also, advanced acquisition protocols like described by Karakatsanis et al. [36] could benefit from CTM.

## Conclusions

The NEMA performance parameters obtained for the mCT Flow system are in concordance with the published values for the predecessor system. No noticeable differences were seen in the contrast recovery as well as in the image quality obtained with sequential and CTM acquisition modes, although the background variability in the phantom measurements was slightly higher when using CTM acquisitions.

## Ethical approval

All procedures performed in studies involving human participants were approved by the regional ethics committee and were in accordance with the 1964 Helsinki declaration.
